# The Impact of Social Media on Public Health and Education: Mechanisms, Vulnerabilities, and Policy Implications

**DOI:** 10.3390/ejihpe16030043

**Published:** 2026-03-12

**Authors:** Keren Dopelt

**Affiliations:** Department of Public Health, Ashkelon Academic College, Ashkelon 78211, Israel; dopelt@bgu.ac.il

## 1. Introduction

In the digital era, the line between evidence-based health information and viral misinformation has become increasingly blurred (Scheufele et al., 2021; Y. Wang et al., 2019). Health misinformation refers to false or misleading health-related claims shared without regard to scientific evidence, regardless of intent (Vraga & Bode, 2020). Social media platforms, while serving as an abundant source of information for billions worldwide, have simultaneously emerged as loci for the dissemination of misleading and inaccurate health content (Y. Wang et al., 2019; Tsao et al., 2021). Algorithm-driven content prioritization, centered on engagement rather than scientific validity, amplifies sensational narratives and facilitates the rapid spread of health misinformation (Feng et al., 2025). Empirical evidence suggests that false information diffuses significantly faster, deeper, and more broadly than truthful content on social networks, largely driven by novelty and emotional engagement (Vosoughi et al., 2018).

The widespread circulation of inaccurate health information across social media platforms constitutes a growing public health concern with tangible behavioral consequences. Exposure to misleading narratives has been associated with vaccination hesitancy, disordered eating patterns, body image dissatisfaction, excessive exercise behaviors, and the consumption of unregulated or potentially hazardous supplements (Pummerer et al., 2022). Beyond influencing individual choices, the high prevalence of health misinformation on social media may undermine evidence-based preventive behaviors and contribute to broader public health risks (Suarez-Lledo & Alvarez-Galvez, 2021).

In response to these challenges, the European Journal of Investigation in Health, Psychology and Education presents this Special Issue on “The Impact of Social Media on Public Health and Education.” The contributions assembled herein not only examine the multifaceted relationship between social media and health behaviors but also critically explore the psychological, educational, and societal mechanisms underlying this phenomenon. By integrating interdisciplinary perspectives, this Special Issue seeks to advance scholarly dialogue and inform future research, policy, and practice. Understanding these dynamics requires examining the mechanisms through which social media actively shapes health behavior.

## 2. How Social Media Shapes Health Behavior

Social media platforms shape health behavior not only by increasing access to information, but by structuring how that information is encountered, interpreted, and trusted (Paul & Headley-Johnson, 2025). Platform personalization systems privilege visibility based on interaction metrics rather than epistemic quality, reinforcing certain narratives and fostering echo chambers in which repeated exposure enhances perceived credibility (Metzler & Garcia, 2024). In such environments, emotionally charged, visually compelling, or sensational health messages are more likely to circulate widely than nuanced, evidence-based guidance (Carmona Pestaña et al., 2025).

Beyond platform-level amplification, the persuasive impact of health content is further shaped by how individuals cognitively process and evaluate information. Users frequently rely on heuristic cues, such as popularity metrics, perceived authenticity, and source attractiveness, when evaluating digital content, rather than systematically assessing scientific evidence (Metzger & Flanagin, 2013). Repeated exposure to similar claims may increase perceived accuracy through familiarity effects, strengthening belief even in the absence of empirical support (Pillai & Fazio, 2021).

In addition to cognitive heuristics, relational dynamics embedded in influencer culture further intensify persuasive effects. Influencers, often lacking formal healthcare training, frequently shape public perceptions of diet, exercise, vaccination, and medical treatments, sometimes more effectively than trained health professionals (Basch et al., 2025; L. Wang et al., 2024). Through parasocial relationships, audiences develop a sense of intimacy and trust, which enhances the persuasive impact. Health advice embedded within lifestyle content may therefore be adopted not because of demonstrated efficacy, but because it aligns with aspirational identities and perceived authenticity (Sokolova & Kefi, 2020).

Together, these structural, cognitive, and relational processes position social media not merely as a communication channel but as a commercialized behavioral health ecosystem (Tu & Li, 2024). Importantly, the same mechanisms that amplify misleading health narratives may also facilitate the dissemination of evidence-based guidance. Social media platforms have demonstrated potential to support health education, promote preventive behaviors, and enhance peer-to-peer support among individuals managing chronic conditions (Moorhead et al., 2013; Naslund et al., 2016). During public health emergencies, digital platforms have been used to rapidly communicate risk information and mobilize communities at scale (Paul & Headley-Johnson, 2025). Systematic reviews suggest that the impact of social media on health behaviors is neither uniformly harmful nor uniformly beneficial, but context-dependent and shaped by platform governance, content credibility, and user engagement patterns.

While social media platforms can disseminate evidence-based guidance, the structural logic governing visibility often prioritizes engagement over accuracy. Engagement-driven architectures are structurally misaligned with population health objectives, as they reward emotional intensity and novelty rather than epistemic reliability.

## 3. Vulnerable Populations and Amplified Risk

While social media influences health behavior across demographic groups, its effects are not distributed equally. Adolescents and young adults are particularly susceptible to the persuasive and normative power of digital health content. Developmental sensitivity to peer evaluation and identity formation increases vulnerability to social comparison processes, especially in visually driven platforms (Avci et al., 2025; Rounsefell et al., 2020). Exposure to curated body ideals, restrictive dietary trends, and extreme fitness regimens has been associated with body dissatisfaction and disordered eating patterns (Dopelt & Houminer-Klepar, 2025; Rounsefell et al., 2020).

Structural inequalities further shape digital vulnerability. Individuals with limited health literacy or reduced access to formal healthcare resources may also face elevated risk. Social media can become a primary source of health information, increasing exposure to misleading or oversimplified claims (Suarez-Lledo & Alvarez-Galvez, 2021). Research suggests that lower eHealth literacy is associated with greater difficulty distinguishing credibility from non-credible online health information (Milanti et al., 2025; Norman & Skinner, 2006). In parallel, communities characterized by historical mistrust toward medical institutions may be more receptive to alternative health narratives circulating online, particularly when communicated through relatable influencers rather than institutional authorities (Dopelt et al., 2023; Jamison et al., 2020).

Individuals managing chronic conditions frequently turn to digital communities for support, experiential knowledge, and validation. While such engagement can enhance empowerment and coping (Naslund et al., 2016), it may also expose users to unverified treatments, anecdotal remedies, or anti-medical discourses. When personal vulnerability converges with algorithmic amplification and parasocial trust, misleading content may become especially compelling, particularly when it resonates with unmet needs or prior skepticism (Cinelli et al., 2021; Lewandowsky et al., 2017).

This commercialized behavioral ecosystem reflects broader commercial determinants of health operating within digital infrastructures. Beyond individual vulnerability, it is also necessary to consider the commercial determinants of health (CDoH) shaping digital environments. While social media is often discussed through the lens of individual choice, these platforms operate within business models that prioritize engagement as a primary commodity, frequently rendering health considerations secondary. In the digital economy, health-related content may function as a byproduct, whereas sustained user attention is the product. For industries promoting health-harming goods, such as ultra-processed foods, alcohol, or tobacco, social media provides highly targeted advertising infrastructures that can circumvent traditional regulatory safeguards. By leveraging detailed user data, commercial actors may identify and exploit psychological vulnerabilities, contributing to the normalization of behaviors detrimental to public health. In this sense, the “environment” in which health behaviors unfold is not only social or psychological but also commercial, structured by corporate incentives embedded within platform design (Gilmore et al., 2023). The attention economy itself increasingly functions as a determinant of health, shaping not only what information circulates, but which health norms become socially dominant. When engagement becomes the primary metric for value, visibility, and, by extension, perceived legitimacy, it may become decoupled from scientific credibility.

## 4. The Role of Health Professionals and Educators

As social media continues to shape how health information is interpreted and acted upon, the role of health professionals and educators within digital discourse has become increasingly consequential. Healthcare institutions and practitioners are already using social media for communication, education, and outreach (Moorhead et al., 2013). However, the accelerating circulation of health information, particularly misinformation, requires more deliberate and strategically designed engagement. In digital environments where visibility often influences perceived credibility, professional silence may allow misleading narratives to gain disproportionate influence (Chou et al., 2018). The transformation of health communication in the digital age has reached a point where disengagement carries tangible consequences.

Healthcare professionals possess the expertise necessary to contextualize risk, interpret scientific uncertainty, and translate complex findings into accessible guidance. Yet traditional communication models, grounded in institutional authority and formal dissemination channels, may be insufficient within fast-paced, algorithm-driven environments (Southwell et al., 2019). In environments structured to reward high-arousal emotional content, nuanced scientific communication struggles to compete with polarizing narratives (Cascini et al., 2022). Effective digital engagement requires not only accuracy but also clarity, relatability, and an understanding of platform dynamics. Bridging the gap between scientific rigor and digital communication norms is therefore essential for maintaining public trust (Larson, 2018).

Educational institutions also play a critical role. Integrating digital health literacy into curricula can equip individuals with the skills needed to evaluate online content, recognize persuasive techniques, and distinguish between evidence-based information and misleading claims (Dopelt et al., 2021; Milanti et al., 2025). Rather than framing social media solely as a risk environment, educational approaches can foster critical engagement and empower users to navigate digital health spaces responsibly.

At the policy level, coordinated strategies are required to address the structural drivers of misinformation. Recent regulatory initiatives, such as the European Union’s Digital Services Act and the World Health Organization’s infodemic management framework, reflect growing recognition of the need for greater transparency, platform accountability, and cross-sector collaboration in digital health governance. International public health bodies have emphasized the importance of infodemic management, transparency, and cross-sector collaboration in mitigating digital health risks (Briand et al., 2023; World Health Organization, 2020). Developing ethical guidelines for health-related content creation, promoting transparent disclosure practices among influencers, and strengthening accountability mechanisms for platform-driven amplification represent key workforce-level avenues for intervention (Bashkin et al., 2021). Such approaches may include graded content labeling for health claims and the downranking of demonstrably harmful misinformation within recommendation systems. They may also involve formalized collaborations with independent fact-checking initiatives, implemented with explicit safeguards for freedom of expression and due process. While regulation alone cannot eliminate misinformation, coordinated professional engagement and evidence-informed policy can mitigate its impact and reinforce public trust in health communication (Bashkin et al., 2022; Dopelt et al., 2023).

## 5. Implications for Research and Policy

Despite the expanding body of research, critical gaps remain in our understanding of how social media shapes sustained health behaviors and policy responses. While the prevalence of health misinformation and its behavioral correlates is well-documented, long-term causal pathways, cumulative exposure effects, and differential susceptibility across populations remain insufficiently understood. Longitudinal and experimental designs are needed to clarify how digitally mediated exposure interacts with psychological vulnerability to shape sustained health behaviors over time.

Research in this domain faces structural constraints. Rapid platform evolution, opaque recommendation systems, and limited data access restrict the ability to examine mechanisms of digital amplification. Without improved access to platform-level data and structured collaboration with technology companies, the development of targeted, evidence-based interventions will likely remain constrained.

Policy responses must therefore move beyond reactive regulation. Effective governance should promote transparency in sponsored health content, enforce clear disclosure standards for influencers, and address the amplification of demonstrably harmful misinformation, while safeguarding freedom of expression. Addressing these challenges requires coordinated engagement across public health authorities, policymakers, educators, researchers, and digital platform stakeholders.

These developments suggest that digital health communication stands at a regulatory and technological inflection point. The rapid integration of artificial intelligence (AI) into content production and recommendation systems introduces an additional layer of complexity. AI-generated health narratives, synthetic influencers, automated bot amplification, and increasingly personalized persuasive messaging may further blur the boundaries between authentic expertise and algorithmically constructed authority. As generative technologies lower the barriers to producing credible-looking health content at scale, traditional markers of trustworthiness become more difficult to assess. This evolution intensifies the urgency of developing governance frameworks that address not only misinformation but the automation of persuasion itself.

## 6. The Role of This Special Issue—A Call to Action

This Special Issue of the European Journal of Investigation in Health, Psychology and Education serves as a platform for researchers, policymakers, educators, and healthcare professionals to critically examine the evolving relationship between social media and public health and education. As digital environments increasingly shape how health information is produced, disseminated, and consumed, coordinated scholarly engagement is essential.

By bringing together interdisciplinary perspectives, this Special Issue seeks to achieve the following:

1. Illuminate the mechanisms through which social media platforms influence health perceptions, norms, and behaviors.
2. Deepen the understanding of the psychological, social, and structural factors that contribute to the spread and persistence of health misinformation.
3. Examine the differential impact of digital health environments across populations, with particular attention to vulnerability and equity.
4. Inform the development of evidence-based communication strategies, educational frameworks, and policy responses.
5. Identify critical gaps in current knowledge and establish priorities for future research and cross-sector collaboration.

The articles included in this Special Issue reflect the multifaceted psychological and behavioral dimensions of social media engagement. Contributions examine how digital exposure shapes emotional processing, sleep patterns, depressive symptoms, body image, aggression, procrastination, loneliness, self-esteem, and vaccine uptake across diverse cultural contexts and age groups. Several studies explore mediating and moderating mechanisms, such as sleep disturbance, psychosocial vulnerability, and contextual influences, highlighting the complex pathways through which social media use translates into measurable health and educational outcomes. Together, these investigations underscore that social media is not merely a communication channel, but a psychologically active environment capable of influencing cognition, emotion, identity formation, and health-related behavior.

By fostering multidisciplinary dialogue, this Special Issue aims not only to analyze the challenges posed by social media but also to highlight its potential as a tool for responsible health promotion, for example, through digitally coordinated vaccination campaigns or online peer-support communities that demonstrably improve treatment adherence. Digital platforms are neither inherently detrimental nor inherently protective. Their public health impact ultimately reflects how they are governed, regulated, and engaged by institutions and users alike.

As algorithmic architectures increasingly shape access to health information, the responsibility to uphold evidence-based guidance extends beyond individual users to institutions, researchers, educators, and policymakers. Social media should no longer be conceptualized solely as a communication infrastructure, but as a powerful commercial determinant of health. The erosion of public trust may partly reflect structural features of digital business models that prioritize engagement-driven amplification over scientific nuance. Addressing the health consequences of digital environments, therefore, requires more than correcting isolated instances of misinformation; it requires sustained attention to platform governance, transparency, and accountability. As digital ecosystems continue to evolve through short-form formats, intensified personalization, and emerging AI-mediated content production, the governance of health information becomes increasingly complex. The future of public health will not be determined only in clinics and classrooms, but within algorithmic systems that govern attention, credibility, and collective perception. Failing to address this structural reality risks leaving population health subordinate to commercial logics of amplification.
